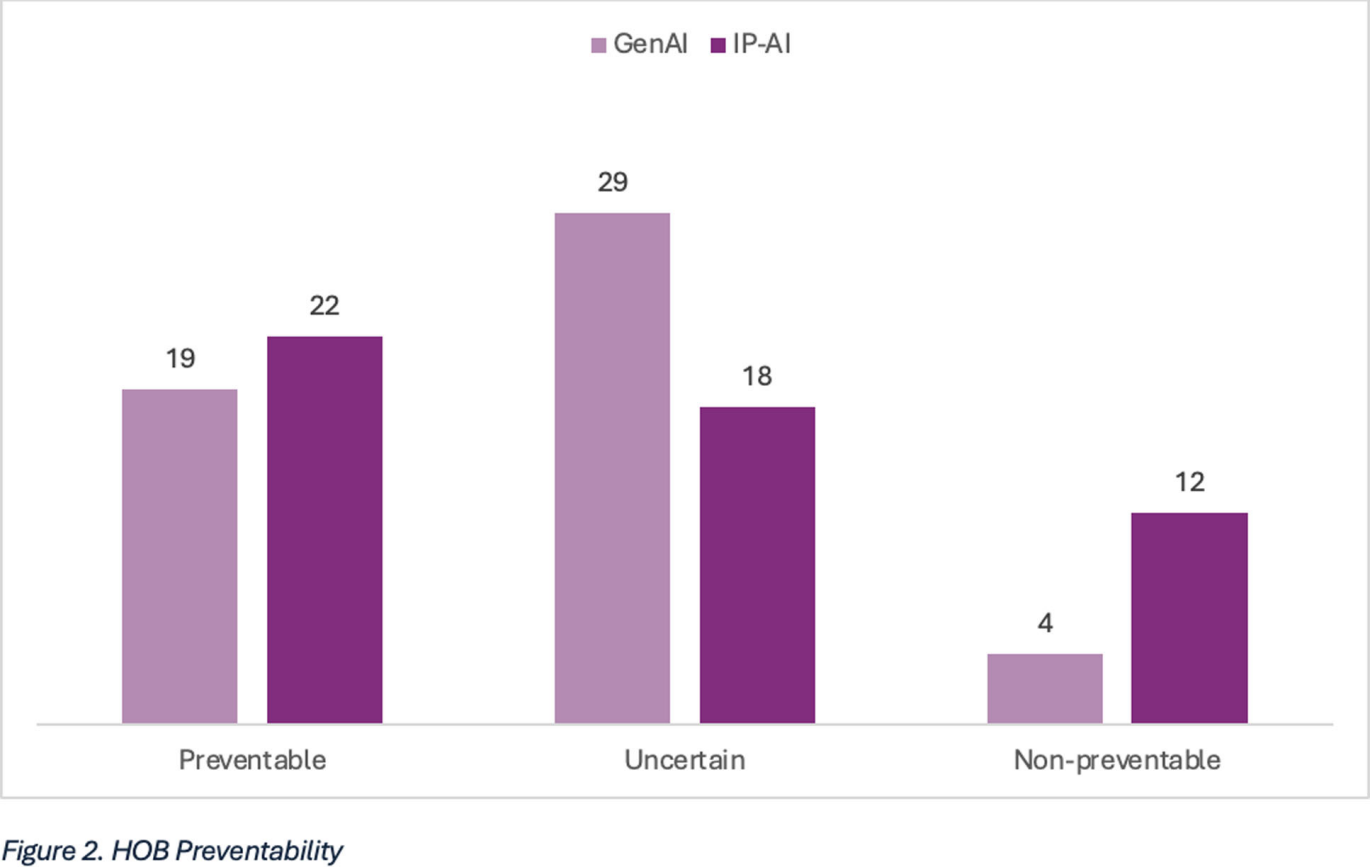# 226 Patterns and Gaps in Mpox Vaccination at NYC Health + Hospitals, a Large Urban Safety Net Health System in New York City, 2022-2025

**DOI:** 10.1017/ash.2026.10728

**Published:** 2026-06-23

**Authors:** Shatha AlShanqeeti, Gaayathri Krishnan, Crisanta Simon, Jonathan Baghdadi, Katherine Goodman, Gregory Schrank, Jessica Holman, Lisa Pineles, Cassie Cunningham Goedken, Christine Firestone, KC Coffey, Westyn Branch-Elliman, Surbhi Leekha, Anthony Harris, Daniel Morgan

**Affiliations:** 1 University of Maryland School of Medicine; 2 University of Maryland; 3 University of Maryland, Baltimore; 4 Center for Access & Delivery Research & Evaluation (CADRE), Iowa City Veterans Affairs Health Care System, Iowa City, IA, USA; 5 Massachusetts General Hospital; 6 University of Maryland Baltimore

## Abstract

**Background:** Hospital onset bacteremia and fungemia (HOB) is under development as a Center for Disease Control and Prevention (CDC) automated digital quality measure. We evaluated the use of generative artificial intelligence (GenAI) in assessing attribution and preventability of HOB. Methods This is a retrospective cohort study of hospitalized patients with positive blood cultures at 11 US Veterans Affairs (VA) hospitals. We included a random sample of positive blood cultures collected after day 3 of admission where admission date is day 1. GenAI (OpenAI GPT-4o) was prompted to provide the most likely source and adjudicate the preventability of HOB using standardized prompts and clinical data (GenAI). IP experts used GenAI output to provide GenAI-assisted determinations of source and preventability of HOB (IP-AI). HOB event preventability was rated by IP expert using a 6-point Likert scale, responses were combined to 3 categories for analysis. We compared initial GenAI with IP-AI determinations of source and preventability. A separate human-only review by a different IP expert was performed (IP-alone) on a random subset to compare time required for review. Results We included 52 HOB events for IP-AI and a subset of 21 for IP-alone. Enterobacterales were the most common organisms (22/52, 42%) followed by Staphylococcus aureus (9/52, 17%). Median duration to HOB event was 12 (IQR 5 – 31) days. GenAI most commonly attributed bacteremia to gastrointestinal (GI) (14/52, 26.9%), followed by genitourinary (GU) (12/52, 23%), central line (CLABSI) (8/52, 15%), and skin and soft tissue infections (SSTI) (7/52, 14%) sources (Figure 1). IP-AI determination of source agreed with GenAI source in 67% (35/52) of cases. Most agreement between IP-AI and GenAI occurred when HOB was attributed to a bone and joint infection (2/2, 100%), followed by GI source (13/14, 93%), SSTI (5/7, 71%), and GU source (7/12, 58%). Most disagreements (17/52, 33%) occurred when IP-AI attribution was to an unknown source (5/17, 29%) or blood culture contamination (3/17, 18%). GenAI adjudication of preventability matched IP-AI adjudication in 20/52 (39%). The median time to complete an IP-AI review was 11.5 (IQR 6 – 20.5) minutes compared to a median of 25 (IQR 13 – 33) minutes for IP-alone. Conclusion IP experts often agreed with GenAI for source of HOB but disagreed with its preventability. GenAI rarely acknowledged unknown source. Using GenAI for HOB detection is faster than human review but must account for differences in preventability.

**Figure:**
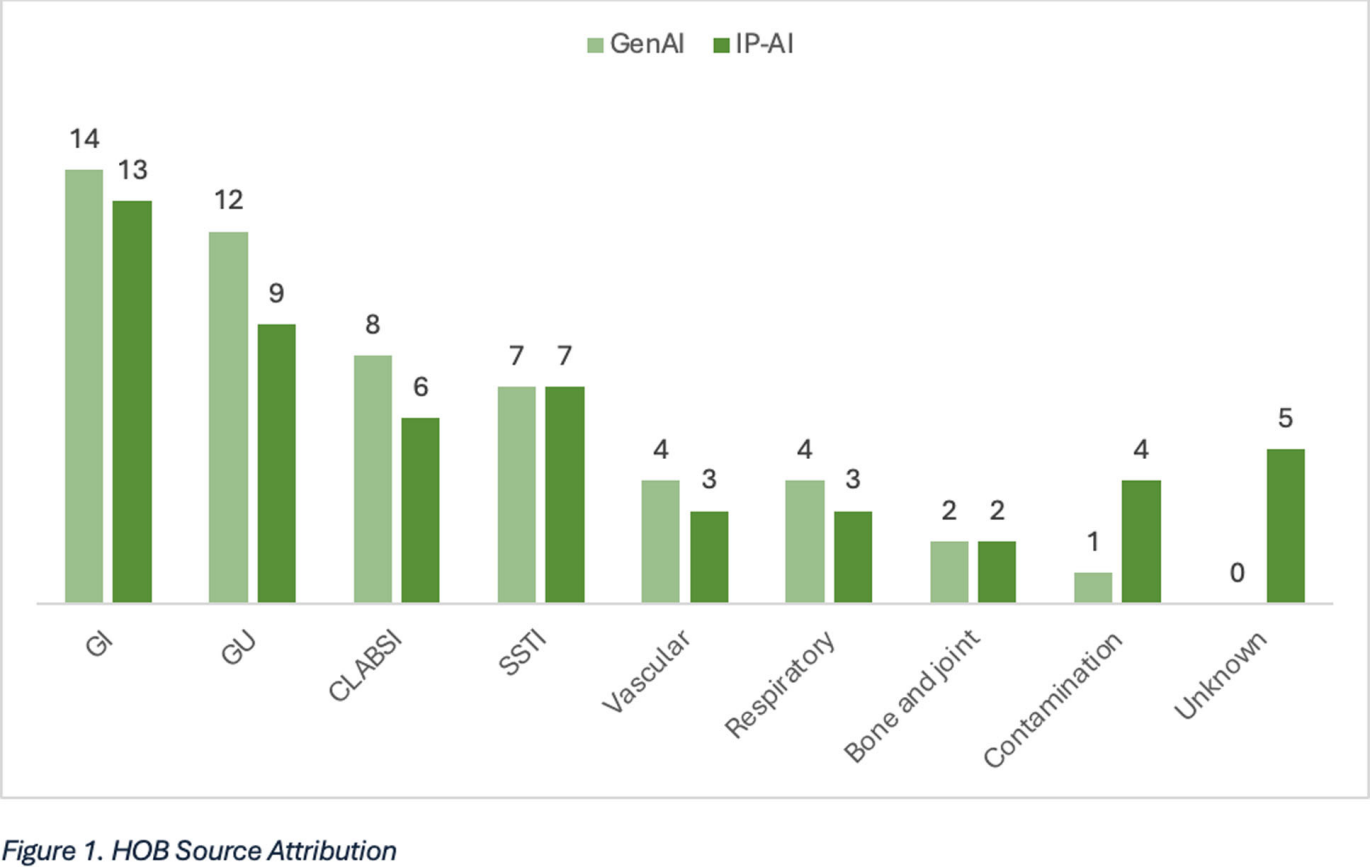

**Figure:**